# Solid-state dye-sensitized solar cells based on ZnO nanoparticle and nanorod array hybrid photoanodes

**DOI:** 10.1186/1556-276X-6-517

**Published:** 2011-09-01

**Authors:** Tao-Hua Lee, Hung-Jue Sue, Xing Cheng

**Affiliations:** 1 Department of Electrical and Computer Engineering, Texas A&M University, College Station, TX 77843, USA; 2 Department of Mechanical Engineering, Texas A&M University, College Station, TX 77843, USA; 3 Polymer Technology Center, Texas A&M University, College Station, TX 77843, USA

**Keywords:** organic photovoltaic, ZnO, nanoparticle, nanorod

## Abstract

The effect of ZnO photoanode morphology on the performance of solid-state dye-sensitized solar cells (DSSCs) is reported. Four different structures of dye-loaded ZnO layers have been fabricated in conjunction with poly(3-hexylthiophene). A significant improvement in device efficiency with ZnO nanorod arrays as photoanodes has been achieved by filling the interstitial voids of the nanorod arrays with ZnO nanoparticles. The overall power conversion efficiency increases from 0.13% for a nanorod-only device to 0.34% for a device with combined nanoparticles and nanorod arrays. The higher device efficiency in solid-state DSSCs with hybrid nanorod/nanoparticle photoanodes is originated from both large surface area provided by nanoparticles for dye adsorption and efficient charge transport provided by the nanorod arrays to reduce the recombinations of photogenerated carriers.

## Background

The rapidly increasing fossil fuel consumption and excessive greenhouse gas emissions have put significant pressure on the already exhaustive global energy demand and needs for environmental protection. The global growing demand for energy and for protecting our environment can potentially be met by solar cell technology. Although the solar cells technology has not yet been in large-scale utilization because of its high cost and insufficient conversion efficiencies in the past, recent advances in nanomaterial and device technologies have offered new opportunities for it to become competitive to fossil fuels. Among the diverse photovoltaic devices, the dye-sensitized solar cells (DSSCs) technology has made enormous progresses and is highly competitive for large-scale commercial fabrication.

DSSCs have emerged as an attractive choice for solar energy harvesting since their invention [1]. The critical component in DSSCs is the photoanode, which is typically composed of a porous TiO_2 _or ZnO nanoparticle film with dye molecules adsorbed onto its surface. To achieve high performance, the photoanode needs to possess a large surface area and good electron transport capability. A TiO_2 _or ZnO nanoparticle film provides a large enough surface area; however, electron transport is difficult because of the need for electrons to hop across neighboring nanoparticles. Moreover, it is well-known that semiconducting particle surfaces are prone to form defects that can act as electron trapping centers. The presence of these surface traps is detrimental to electron transport because trapping/detrapping events are unavoidable during electron diffusion through the disordered nanoparticle network [2-4]. By altering the morphology of the photoanode, electron transport pathways may be designed to improve electron collection.

DSSCs based on dense ZnO nanowire/nanorod arrays have been reported to exhibit improved electron transport efficiency [5]. Intensity-modulated photovoltage and photocurrent spectroscopies have revealed that photoanode based on ZnO nanorod arrays exhibits two orders of magnitude faster electron transport while retaining similar electron recombination time compared to photoanodes based on nanoparticles [6]. However, the photocurrents and the efficiencies of the nanowire/nanorod-based DSSCs are limited by insufficient surface area for dye adsorption [5]. To further improve the performance of DSSCs, various ZnO structures, such as branch structure [7], nanoflower [8], and hybrid nanowire/nanoparticle [7,9,10] have been employed as the photoanodes to achieve fast electron transport while maintaining a large surface area for dye coating.

Despite their high efficiency, DSSCs based on liquid electrolyte have reliability issues caused by the liquid redox electrolyte. Device instability and the need for good device packaging have become major hurdles for commercial application of DSSCs [11]. Furthermore, liquid electrolyte based solar cells cannot be easily fabricated into multicell modules [12]. One way to address this manufacturing difficulty is to replace the liquid redox electrolyte by a solid-state hole transport material, typically a p-type conjugated polymer. Recently, many attempts have been made by using different hole transport materials, such as OMeTAD [13,14], pentacene [15], poly(triphenyldiamine) [16], polythiophene [17], and poly(3-hexylthiophene) (P3HT) [18], along with dye-loaded porous nanoparticle films. Solid-state DSSCs with ZnO nanorod arrays as photoanodes and different conjugated polymers as hole transport material have also been reported with efficiencies of ~0.20% [19,20].

In this work, we, to the best of our knowledge, for the first time explore the use of ZnO nanorod and ZnO nanoparticle hybrid electrodes for solid-state DSSCs. We fabricated solid-state DSSCs by using ZnO nanoparticles to fill the interstitial voids between ZnO nanorod arrays as the photoanode, hoping to further improve the efficiency of the solid-state DSSC device. The ZnO nanorod arrays serve as direct pathways for fast electron transport, and the ZnO nanoparticles filled in the interstitial space of ZnO nanorods offer a large surface area for dye adsorption. Using this hybrid nanorod-nanoparticle structure, a significant improvement in performance has been achieved. The effects of the ZnO photoanode morphology on the solid-state DSSC's performance are discussed.

## Results and discussion

**Figure 1:**
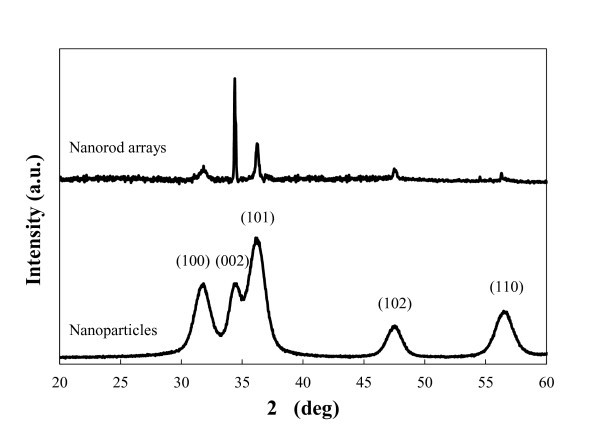
**X-ray diffraction patterns of the ZnO nanorod arrays and ZnO nanoparticles**.

The degree of crystal orientation of ZnO nanorod arrays and nanoparticles were determined by X-ray diffraction (XRD) spectrum as shown in Figure 1. The dominant peak for randomly oriented ZnO powders is at 36.2° and corresponds to (101) plane. For nanorod arrays, the strong diffraction peak seen at 34.4° corresponds to the ZnO (002) plane. The enhanced (002) peak indicates the ZnO nanorods grow along *c*-axis and perpendicular to the substrate.

**Figure 2:**
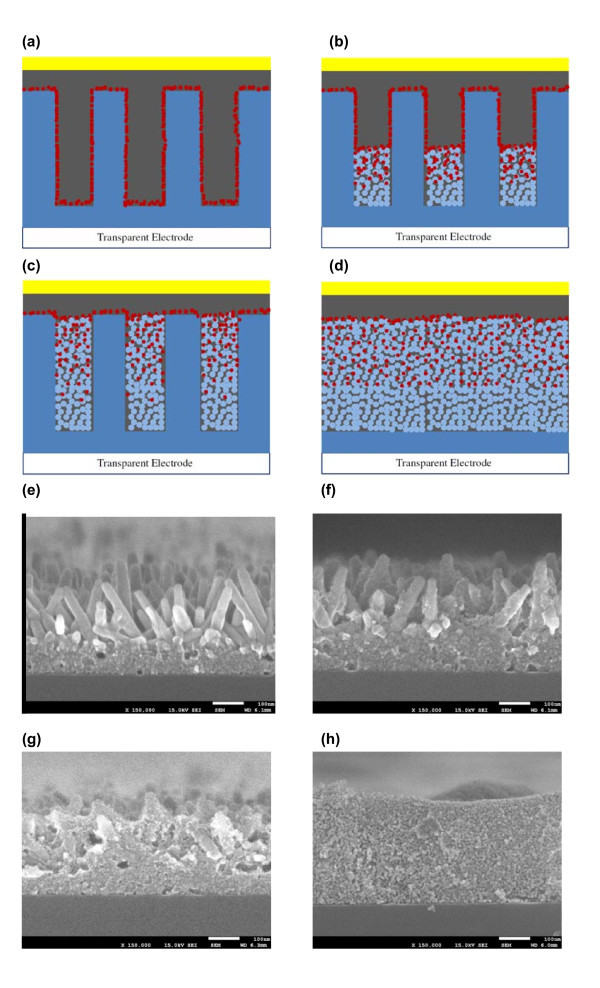
**Schematics and FE-SEM images for solid-state DSSCs with different photoanode morphologies**. Schematics of device based on (**a**) nanorod array photoanode (sample 1), (**b**) nanorod array photoanode with nanoparticles partially filling the interstices (sample 2), (**c**) nanorod array photoanode with nanoparticles fully filling the interstices (sample 3), (**d**) nanoparticle photoanode (sample 4). Blue rectangles represent ZnO nanorods, blue dots represent ZnO nanoparticles, red dots represent N3 dyes, the gray film represents P3HT, and the yellow film represents gold. FE-SEM images of photoanodes for (**e**) sample 1, (**f**) sample 2, (**g**) sample 3, and (**h**) sample 4. The scale bar is 100 nm for all SEM images.

The schematic and field emission scanning electron microscopy (FE-SEM) images of solid-state DSSCs based on four different ZnO photoanode morphologies are shown in Figure 2. Figure 2a shows the schematic of the solar cell based on ZnO nanorod arrays, denoted as sample 1. Sample 2 contains a hybrid ZnO structure in which ZnO nanorod arrays are partially filled with ZnO nanoparticles (Figure 2b). Sample 3 possesses a hybrid ZnO structure in which the interstitial voids between nanorod arrays are fully filled by ZnO nanoparticles (Figure 2c). The difference between sample 2 and sample 3 is the amount of nanoparticles in the interstices between the nanorods. Sample 4 is a photovoltaic device based on ZnO nanoparticles alone (Figure 2d). Figure 2e, f, g, h shows the corresponding FE-SEM images of ZnO photoanodes before dye loading. The average diameter of ZnO nanoparticles is 5 nm. The thickness of the ZnO seed layer for nanorod growth is about 100 nm. ZnO nanorods have a diameter in the range of 30 to 40 nm and are about 250 nm in length. The thickness of the ZnO thin film in sample 4 is kept the same as the length of ZnO nanorods in samples 1 to 3. Solar cells with the four photoanode morphologies are investigated to illustrate the factors that affect the solar cell performance.

Electron transport is much more efficient in single crystal nanorods than in particulate thin films. First, there is a direct pathway for electrons to reach the electrode through nanorods. In addition, nanorods have smaller surface-to-volume ratio compared to nanoparticles, thus possessing fewer surface defect states that trap electrons. Therefore, electrons have a much higher mobility and can travel through nanorods tens to hundreds times faster than that through thin films composed of nanoparticles [5,6,21]. Efficient electron transport helps shorten the time needed for electron to travel to the electrode, thus lowering probability of the recombination loss of electrons.

However, ZnO photoanodes based on nanorod arrays suffer from limited surface area that can absorb dye molecules for efficient light harvesting when compared to the electrodes based on nanoparticles. By filling the interstitial space between nanorods with 5-nm-size ZnO nanoparticles, the total surface area of ZnO photoanodes can be greatly increased. Light absorption of the device can thus be increased without compromising efficient electron transport of ZnO nanorod arrays. Furthermore, compared to more than thousand times hopping in a particulate thin film, electrons injected from dye molecules into nanoparticles can reach ZnO nanorods and then the electrode, just by hopping across a few nanoparticles [7,22]. By minimizing the number of interparticle hoppings, the carrier recombination can be greatly reduced. Consequently, it is anticipated that with increasing number of dye molecules on ZnO surfaces and with less recombination of electron-hole pairs by using the hybrid ZnO nanorod-nanoparticle photoanode morphologies shown in Figure 2b, c, large photocurrents can be generated while maintaining a high open-circuit voltage.

**Figure 3:**
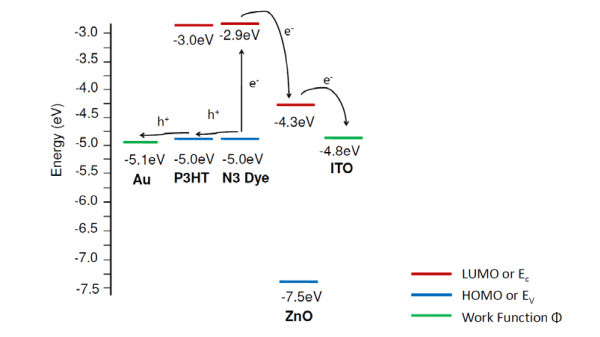
**A schematic of the energy level diagram of the FTO/ZnO/N3/P3HT/Au device**.

In this study, we use N3 dye as the light-absorbing material for the device. Even though P3HT can also function as the light-absorbing component, its main function is to transport holes in the device. Two layers of P3HT were spin-coated on top of the dye-loaded ZnO photoanode. The first spin-coating with diluted P3HT solution provides good wetting of the ZnO photoanode, while the second spin-coating with concentrated P3HT solution deposits a thick enough film to smooth out the surface irregularity of ZnO photoanode to prevent electrode shorting. Without spin-coating the diluted P3HT as the first layer, voids can be formed between ZnO photoanode. Figure 3 shows the expected energy levels of the materials from reported values in literature [23-25]. The conduction band of ZnO is lower than the lowest unoccupied molecular orbital (LUMO) level of N3, enabling electron transfer from N3 to ZnO. Although the LUMO level of the P3HT is similar to that of N3, the difference between the LUMO level of P3HT and the N3 dye is much smaller than the difference between the conduction band of ZnO and the LUMO level of the N3 dye. Accompanied with very low electron mobility in P3HT, majority of electrons are expected to be injected from N3 to ZnO.

**Figure 4:**
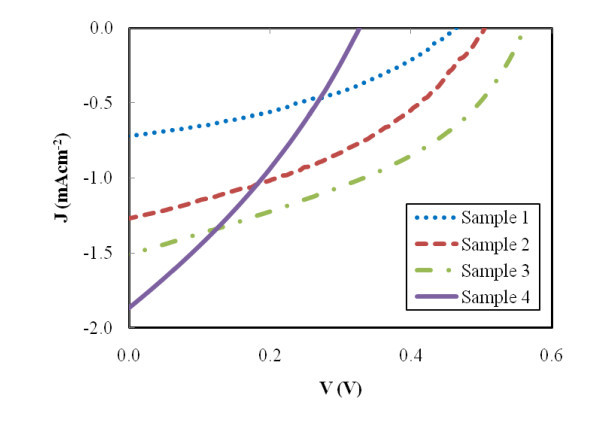
**Current-voltage characteristics of FTO/ZnO/N3/P3HT/Au devices based on different ZnO photoanodes**.

ZnO solar cells with structures described in Figure 2 were characterized by measuring the current density-voltage (*J*-*V*) behavior under air mass (AM) 1.5 condition. Figure 4 shows typical *J*-*V *curves of the devices with different morphologies. Table 1 summarizes short-circuit currents (*J*_sc_), open-circuit voltages (*V*_oc_), fill factors (FF), and overall energy conversion efficiencies (*η*) for devices based on various photoanode morphologies.

**Table 1:** Device parameters for solid-state DSSCs with different ZnO morphologies based on three batches of samples

Sample	*J*_sc _(mA cm^-2^)	*V*_oc _(V)	FF	*η *(%)
1	0.72 ± 0.12	0.46 ± 0.05	0.38 ± 0.02	0.13 ± 0.02
2	1.27 ± 0.16	0.50 ± 0.07	0.39 ± 0.01	0.25 ± 0.01
3	1.52 ± 0.04	0.56 ± 0.01	0.40 ± 0.01	0.34 ± 0.02
4	1.86 ± 0.11	0.32 ± 0.03	0.31 ± 0.03	0.19 ± 0.01

**Figure 5:**
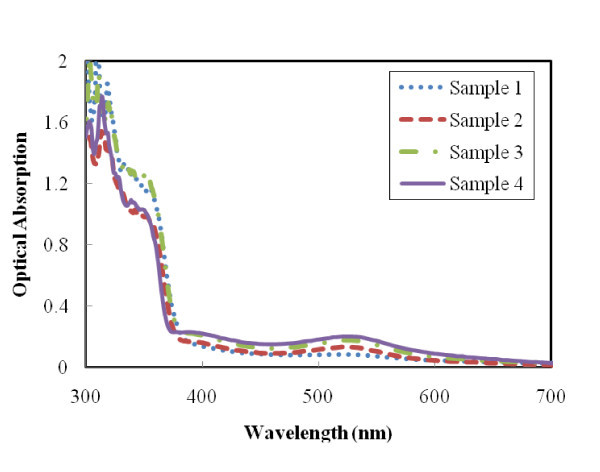
**Optical absorption of ZnO photoanodes with 30-min dye loading**.

*J*_sc _is determined by the initial number of photogenerated carriers and the injection effectiveness of electrons from dye molecules to metal oxide [26]. Since the devices are composed of the same materials, it is assumed that the electron injection efficiency from N3 dye to ZnO photoanode is the same for all morphologies. Thus, the differences in *J*_sc _values from the devices are due to the initial number of photogenerated carriers, which is proportional to the light-harvesting capability of the dye-loaded ZnO electrode. Optical absorption spectra of dye-loaded ZnO photoanodes show the variation in light absorption capability of different ZnO structures. In Figure 5, all the samples exhibit an intrinsic absorption with similar absorption intensity below 380 nm, which is due to the band gap absorption in ZnO. On the other hand, significant variation in light absorption capability at wavelengths above 380 nm is mainly originated from the density of the dye molecules on the ZnO surfaces and is related to the photoanode morphology. It should be noted that the absorption peak of N3 dye is centered at around 520 nm. Among four different ZnO thin film morphologies, the device based on sample 4 has the highest absorption intensity, followed by sample 3, sample 2, and sample 1. This absorption intensity corresponds to the total internal surface area of the ZnO film. The optical spectra illustrate that more effective photon capturing is achieved in the visible light region with larger total surface area of the ZnO photoanode because more N3 dyes can be adsorbed onto the film. With the largest internal surface area and the highest amount of N3 dye, sample 4 has the highest *J*_sc_, followed by the devices based on samples 3, 2, and 1. Their corresponding current densities are 1.86, 1.52, 1.27, and 0.72 mA cm^-2^, respectively.

Sample 1 has a *V*_oc _of 0.46 V compared to 0.32 V for sample 4. The increase in the *V*_oc _indicates that the electron-hole recombination rate is lower in sample 1 than in sample 4 [27]. This is possibly resulted from differences in electrical properties between ZnO nanoparticles and nanorods, and from the faster electron transport in nanorods. With even higher *V*_oc _from sample 2 and sample 3, which are 0.50 and 0.56 V, respectively, it is anticipated that nanorods do improve electron transport compared to porous films composed of nanoparticles. In sample 2 and sample 3, electrons generated from dye-coated nanoparticles adjacent to nanorods need only one single hop to reach nanorods. As for electrons generated from other dye-coated nanoparticles, the number of hops becomes more than one, but this number of hops is still much less than that in sample 4. As a result, nanorods provide efficient transport pathways for electrons coming from surrounding nanoparticles, which reduces the total travel distance for electrons, and thus the chances of electron recombination during transport. Another possible reason is that by filling the voids between nanorods with nanoparticles, the amount of light absorption by P3HT is reduced. Light absorbed in P3HT generates excitons with short diffusion lengths, and most of the excitons will eventually recombine if they are not located very close to the P3HT-ZnO interface. In samples 2 and 3, fewer excitons are generated in P3HT because the P3HT layer is located behind the dye-loaded ZnO nanoparticles. This will generate more useful photo-carriers and increase *V*_oc_.

The fill factors of samples 2 and 3 are 0.39 and 0.40, respectively, which are approximately the same as the FF of sample 1. The FFs of the devices based on hybrid photoanodes are higher than that of the nanoparticle-based device, which is only 0.31. Again, this indicates that nanorod arrays serve as efficient transport pathways for electrons, and the parasitic losses in the devices containing nanorod arrays are lower than those containing nanoparticles only. As a result, sample 3 has the highest overall energy conversion efficiency, followed by sample 2, sample 4, and sample 1 with their respective efficiency of 0.34%, 0.25%, 0.19%, and 0.13%.

**Figure 6:**
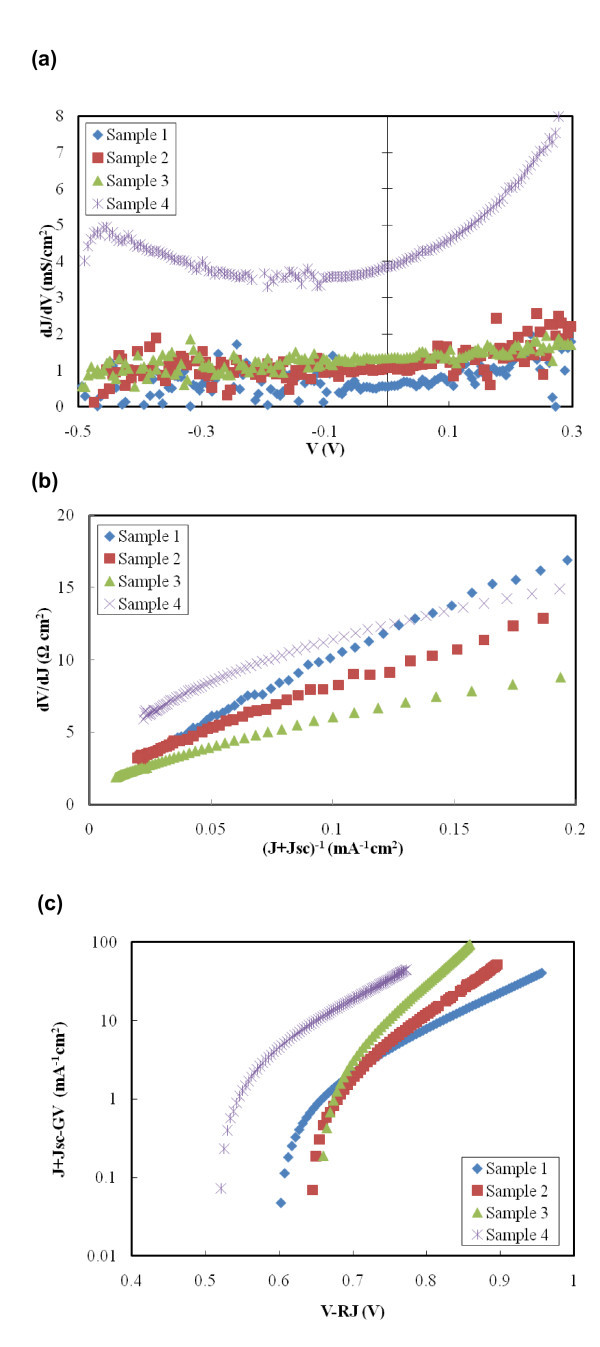
**Plots -for diode parameter determination**. (**a**) d*J*/d*V *versus *V *for shunt conductance (*G*) characterization. (**b**) d*V*/d*J *versus (*J *+ *J*_sc_)^-1 ^for the determination of serious resistance (*R*) and diode ideality factor (*A*). (**c**) *J *+ *J*_sc _- *GV *versus *V *- *RJ *for the determination of reverse saturation current (*J*_o_).

While *J*_sc_, *V*_oc_, FF, and *η *are well-accepted indicators for solar cell performance, additional information can be obtained by analyzing the entire *J*-*V *curve. We analyzed the measured *J*-*V *characteristics using the lumped circuit model for solar cell and find the diode parameters following the approach described elsewhere [27,28]. The current equation for a solar cell device under illumination can be expressed as:

(1) $$J=J_{\text{o}}\exp\left[\frac{q}{AkT}(V-RJ\right]+GV-J_{\text{L}}$$

where *J *and *V *are the diode current density and voltage, *J*_o _is the diode reverse saturation current density, *q *is the electron charge, *A *is the diode quality factor, *k *is the Boltzmann constant, *T *is temperature, *R *is the series resistance, *G *is the shunt conductance, and *J*_L _is the photocurrent density. Diode parameters can be determined from the standard *J*-*V *curve shown in Figure 4 along with plots shown in Figure 6.

The value of *G *can be extracted from Figure 6a, where d*J*/d*V *versus *V *is plotted. For an efficient solar cell, the value *G *should be as small as possible to prevent internal leakage of current through the cell. This leakage limits the FF of the device. Calculated values of *G *are listed in Table 2. Sample 1 has the lowest value of *G*, which equals to 0.5 mS/cm^2^. With increasing amount of nanoparticles used in the device, the value of *G *increases. As a result, sample 4 gives the largest value of *G*, 3.8 mS/cm^2 ^and has the largest amount of leakage current through the cell.

**Table 2:** List of curve-fitting diode parameters for solid-state DSSCs with different ZnO morphologies

Sample	*G *(mS cm^-2^)	*A*	*R *(Ω cm^2^)	*J*_o _(mA cm^-2^)
1	0.5	3.72	1.06	1.92 × 10^-3^
2	1.1	2.56	1.93	6.67 × 10^-5^
3	1.3	1.95	1.45	4.01 × 10^-6^
4	3.8	3.07	4.41	4.03 × 10^-3^

By plotting d*V*/d*J *versus (*J *+ *J*_sc_)^-1 ^in Figure 6b, the value of series resistance can be obtained. Same as *G*, for an efficient solar cell, *R *should be as small as possible. From Table 2, sample 1 has the lowest value of *R*, 1.06 Ω cm^2^, and sample 4 gives the largest value of *R*, 4.41 Ω cm^2^.

The ideality factor *A *and the reverse saturation current *J*_o _can help understanding the recombination mechanism of the devices. When *A *is close to 1, the limiting recombination mechanism is the recombination in the charge neutral region. When *A *is close to 2, the limiting recombination mechanism is the recombination in the space-charge region. When *A *is larger than 2, additional recombination mechanisms are incorporated [27]. If we consider values for *A *calculated from Figure 6b, as listed in Table 2, samples 1, 2, and 4 all have the value of *A *larger than 2 and the ideality factor is as high as 3.72 for sample 1. In devices based on these three photoanode morphologies, tunneling recombination for trapped electrons and holes is a significant recombination mechanism. ZnO nanoparticle surfaces and disordered P3HT thin film are full of defects that behave as traps for charges. Trapped electrons have higher chances of geminate recombination with trapped holes by tunneling back to the original molecules [29]. The value of *A *for sample 3 is only 1.95, which suggests that space-charge region recombination is more significant than tunneling recombination in this sample. The fast removal of electrons in ZnO nanoparticles through nanorod arrays may be the reason for low tunneling recombination.

Figure 6c shows a semi-logarithmic plot of *J *+ J_sc _- *GV *against *V *- *RJ*. The value of *J*_o _is again calculated from the plot and is listed in Table 2. For sample 1 and sample 4, *J*_o _= 1.92 × 10^-3 ^and 4.03 × 10^-3^, respectively. For samples 2 and 3, *J*_o _decreases by two to three orders of magnitude to 6.67 × 10^-5 ^and 4.01 × 10^-6^, respectively. Overall *J*_o _decrease is due to decreased defect density and less carrier recombinations.

Although the values of *G *and *R *from sample 3 are not the lowest ones, they are in an acceptable range compared to the other three samples. Sample 3 has the lowest *A *and *J*_o _as a result of having the lowest carrier recombination rate in all samples. By analyzing the values of *G*, *R*, *A*, and *J*_o_, it is reasonable to state that sample 3 gives the best performance and this agrees with the results from *η*. It is also reasonable to suggest that by using nanorod arrays longer than 250 nm as scaffold for the device may bring even higher efficiency. With longer nanorods, more interstitial space can be occupied by ZnO nanoparticles. Thus, more dyes can be loaded onto the ZnO surface. At the same time, though the length of the nanorod arrays is different, the distance between each ZnO nanorod is still in the similar range and charge recombination can be reduced efficiently.

Despite the efficiency improvement with the use of hybrid photoanodes, the solar cell efficiencies obtained in this work are still low compared to similar devices made of TiO_2_. Lower efficiency is almost universally observed in ZnO-based solar cells in published work, so properties directly related to ZnO nanomaterials are most likely the efficiency-limiting factors. These could be high-density defects on the surfaces of ZnO nanomaterials, which trap charges and lead to step-wise electron-hole recombination through mid-bandgap defect energy levels. Additionally, oxygen molecules absorbed at the surface of ZnO nanomaterials can serve as efficient exciton quenching centers. Further experiments are needed to investigate those factors and to shed light on the root cause of lower efficiency in ZnO-based solar cells.

## Conclusions

Solid-state DSSCs based on various morphologies of ZnO photoanodes with N3 dye as the light-absorbing material and P3HT as the hole transport material have been fabricated. The effect of the morphology of the ZnO photoanodes has been investigated. Short-circuit current increases with the amount of ZnO nanoparticles in the photoanode due to large surface area for dye loading. Compared to pure nanoparticle photoanode, *V*_oc _increases with the presence of ZnO nanorod arrays due to faster electron transport and less charge recombination. The overall conversion efficiency of the solid-state DSSC based on ZnO nanorod arrays is 0.13%. By fully filling the interstitial voids of the nanorod arrays with 5-nm-size ZnO nanoparticles, the device efficiency increases significantly to 0.34%. Analysis from diode parameters shows the values of *A *and *J*_o _decrease in solid-state DSSCs based on the hybrid ZnO photoanodes. This suggests that devices based on hybrid ZnO photoanodes have lower charge recombination rate. To improve the efficiency of solid-state DSSCs based on hybrid ZnO photoanodes, further quantitative investigations on charge-trapping defects and carrier recombination rates from various mechanisms in ZnO nanoparticles and ZnO nanorod arrays are still needed.

## Methods

### ZnO nanoparticle synthesis

Colloidal ZnO nanoparticles with nearly uniform diameters of 5 nm were prepared by hydrolyzation process. This method has been previously reported elsewhere [30], only a brief summary is given here. Sixteen mmol of KOH was dissolved in 150 ml methanol at 60°C with refluxing and stirring for 5 min, followed by addition of 8 mmol of zinc acetate dihydrate (Zn(Ac)_2 _2H_2_O) in 50 ml methanol. This mixture was refluxed and stirred at 60°C for 2 h. The ZnO colloids were then concentrated from 200 ml to 20 ml at 60°C by rotary evaporation under vacuum. After adding 100 ml of hexanes and 20 ml of isopropyl alcohol, the mixture was kept at 0°C overnight. ZnO precipitate was then redispersed in methanol with the removal of the supernatant.

### Device fabrication

Fluorine-doped tin oxide glass substrates (FTO, 15 Ω/square) were cleaned by acetone and isopropyl alcohol and coated with a thin layer of ZnO nanoparticles by drop-casting in methanol and annealed at 300°C for 10 min. ZnO nanorods were grown by immersing seeded substrates in aqueous solutions containing 0.05 M zinc nitrate hexahydrate (Zn(NO_3_)_2 _6H_2_O) and 0.05 M methenamine (C_6_H_12_N_4_) at 95°C for 90 min. Subsequently, the nanorod array thin films were rinsed with deionized water and dried in air at the same temperature. Formation of the ZnO nanoparticles in the interstices of the ZnO nanorod arrays was carried out by drop-casting low concentration ZnO nanoparticles in methanol. For nanoparticle photoanodes, ZnO nanoparticles were drop-casted on the seeded substrates to meet the length of nanorod arrays. After annealing at 300°C for 30 min, zinc oxide photoanodes were sensitized in a 0.5 mM solution of RuL_2_(NCS)_2_, L = 2,2'-bibyridyl-4,4'-dicarboxylic acid (N3 dye) in ethanol at room temperature for 30 min. Then, the dye-adsorbed ZnO thin films were immersed in ethanol to remove excess dyes. Regioregular poly(3-hexylthiophene) (P3HT) was dissolved in 1,2-dichlorobenzene and used as hole transport material. A thin layer of P3HT was spin-coated onto dye-loaded photoanodes with a concentration of 0.5 mg/ml at 200 rpm. Another layer of P3HT was then spin-coated onto the films with a concentration of 20 mg/ml at 800 rpm. After spin-coating, devices were annealed in a vacuum oven at 150°C for 30 min. Finally, 100-nm-thick gold contacts were thermally evaporated through a shadow mask. The active area of the devices was 2.25 mm^2^.

### Solar cell characterization

The crystal orientation of nanorod arrays and nanoparticles were recorded by an XRD (Bruker D8 Discover with Cu Kα radiation operated at 40 kV, 40 mA; Bruker AXS, Inc., Madison, WI, USA). The morphologies of the photoanodes were characterized using FE-SEM (JEOL JSM-7500F operated at 10 KeV; JEOL, Ltd., Tokyo, Japan). To evaluate the light absorption of the dye-loaded ZnO photoanodes, UV-vis-NIR (Hitachi U-4100 UV-vis-NIR spectrophotometer; Hitachi, Ltd., Tokyo, Japan) spectra were acquired in absorption mode. To evaluate solar cell performance, the fabricated devices were illuminated under a standard solar simulator at AM 1.5 condition (100 mW/cm^2^) with current density-voltage (*J*-*V*) characteristics acquired by a Keithley 2400 sourcemeter (Keithley Instruments, Inc., Cleveland, OH, USA).

## Competing interests

The authors declare that they have no competing interests.

## Authors' contributions

TL participated in the design of the study, carried out the experiments, collected data, performed data analysis, and drafted the manuscript. HS participated in the design of the study and data analysis, and helped to draft the manuscript. XC conceived the study, participated in its design and coordination, and helped to draft the manuscript. All authors read and approved the final manuscript.
